# A Variable Stiffness System for Impact Analysis in Collaborative Robotics Applications with FPGA-Based Force and Pressure Data Acquisition

**DOI:** 10.3390/s25133913

**Published:** 2025-06-23

**Authors:** Andrea D’Antona, Saverio Farsoni, Jacopo Rizzi, Marcello Bonfè

**Affiliations:** Department of Engineering, University of Ferrara, 44121 Ferrara, Italy; saverio.farsoni@unife.it (S.F.); jacopo.rizzi@unife.it (J.R.); marcello.bonfe@unife.it (M.B.)

**Keywords:** stiffness, robotics, interaction, safety, FPGA, force sensor, impact

## Abstract

The integration of robots into collaborative environments, where they physically interact with humans, requires systems capable of ensuring both safety and performance. This work introduces the development of a Variable Stiffness Impact Testing Device (VSITD), designed to emulate physical human–robot interaction by replicating biomechanical properties such as muscle elasticity and joint compliance. The proposed system integrates a Variable Stiffness Mechanism (VSM) with a multi-sensor configuration that includes a high-resolution Force Sensitive Resistors (FSR) matrix, piezoelectric load cells, and an FPGA-based acquisition unit. The FPGA enables fast acquisition of contact forces and pressures, while the mechanical stiffness configuration of the VSM can be rapidly reconfigured to simulate a wide range of impact scenarios. The device aims to validate compliance with the standard ISO/TS 15066 safety standard of robotic work cell in the context of collaborative application. The modularity and flexibility of the VSITD make it suitable for evaluating a wide range of collaborative robotic platforms, providing a reliable tool for pre-deployment validation in shared workspaces. By combining real-time sensing with adaptable stiffness control, the VSITD establishes a new benchmark for safety testing in human–robot collaboration scenarios.

## 1. Introduction

The growing integration of robots into human-centric environments requires advanced technological solutions that ensure safe, adaptive, and effective physical interactions. In this context, the proposed Variable Stiffness Impact Testing Device (VSITD) leverages a Variable Stiffness Mechanism (VSM) to replicate biomechanical properties of the human body—such as joint compliance and muscle elasticity—thus enhancing the safety and realism of collaborative human–robot interaction (HRI) scenarios [1,2]. The ability to dynamically modulate stiffness provides a critical advantage when validating robotic systems operating in proximity to humans, especially where high precision and safe contact are essential.

The VSITD is a fully integrated platform that combines variable mechanical compliance with hybrid sensing and high-speed data acquisition. The system merges a VSM with a custom sensing architecture composed of a 32×32 Force Sensitive Resistors (FSR) matrix, piezoelectric load cells, and a Field Programmable Gate Array (FPGA). The FPGA is employed not for closed-loop real-time control but to enable rapid and repeatable acquisition of contact forces and pressure data across a wide spatial domain, thereby facilitating stiffness adaptation.

The core elements of the VSITD are the following:

- FSR matrix: A high-resolution sensor grid that captures spatial pressure distribution and localizes contact areas with fine granularity.
- Load cells: Piezoelectric force sensors used to measure point-specific force amplitudes with high sensitivity and temporal resolution, complementing the pressure mapping provided by the FSR matrix.
- FPGA: A hardware platform for high-speed, parallel acquisition, and processing of sensor signals, supporting rapid reconfiguration of stiffness and high-throughput data streaming [3].

This sensor fusion strategy allows the system to achieve comprehensive contact characterization. The FSR matrix delivers valuable spatial resolution for contact area estimation [4,5], while the load cells enhance accuracy in absolute force measurement. Together, these components enable reliable testing of robotic platforms under conditions that closely mimic human biomechanics.

A central objective of the VSITD is the validation of collaborative robotic systems in compliance with safety standards, such as ISO/TS 15066 [6,7,8], which define limits for forces and pressures during physical human–robot contact [9]. Through high-resolution pressure mapping and stiffness adaptation, the device can verify that forces remain within permissible thresholds, even in unexpected contact events [10].

Moreover, the modular design of the VSITD allows for its integration with a broad range of robotic platforms, from assistive technologies to industrial collaborative arms. Its combination of fast reconfigurability, hybrid sensing, and standard compliance positions it as a versatile tool for HRI testing and certification [11].

The structure of this paper is as follows: Section 2 reviews the related work; Section 3 describes the mechanical design and stiffness control mechanism of the VSM; Section 4 details the sensing and acquisition electronics; Section 5 presents the experimental validation; and Section 6 concludes with a summary and future work directions.

## 2. State of the Art

In collaborative robotics, ensuring safety, respecting the normative ISO15066, during human–robot interactions is a critical concern. To achieve this, mechanical devices that simulate human responses, such as biofidel measuring devices, are often employed as presented in [12]. These devices are used as safety heads or simulators to assess and validate the forces and impacts experienced by humans during collaboration with robots. The biofidel device, for example, plays a key role in evaluating safety parameters in work environments where robots are equipped with Power and Force Limiting (PFL) functions, ensuring that interactions remain within safe limits. Such devices are essential for improving the reliability and safety of collaborative robotic systems.

Systems using Fujifilm Prescale Films (FPF) with similar purpose are used in the project of [13]. They introduced the Dynamic Impact Testing and Calibration Instrument (DITCI), a tool designed to assess the impact forces and risks associated with robot–human interactions. DITCI offers adjustable settings for impact forces and foundation stiffness, allowing researchers to simulate various injury scenarios without the need for expensive biological testing. The system supports a wide range of sensors and impact tools, ensuring compatibility with real-world manufacturing environments and safety standards. Data collected through impact motion, force, and torque measurements are used to create models that predict the effects of robot impacts on human tissues. One of the key applications of this tool is the testing of biosimulant human tissue artifacts, which simulate the deformation of soft tissues, such as the abdomen, in response to robot impacts. This approach allows for a more affordable and ethical alternative to traditional human-based testing, offering valuable insights into the severity of injuries that may occur during industrial robot operations. DITCI also provides a means of verifying the effectiveness of existing safety protocols, ensuring that robot tools and control modes are safe for human operators. The technology is particularly beneficial for small manufacturing companies with limited access to advanced safety equipment and resources.

The technology is particularly advantageous for small manufacturing enterprises with limited access to advanced safety systems and resources, where reliance on human operators introduces substantial limitations, such as post-hoc analysis and the lack of continuous, real-time data acquisition. The work presented in [14] addressed this issue by analyzing the power and force-limited mode of robots, which is crucial to prevent injuries during close interactions. It highlights the need for precise measurements of force and pressure during human–robot contact as part of the safety assessment process. However, the precise procedure for reliably measuring these forces is still not fully defined, making it challenging for system integrators to self-assess the safety of their collaborative robotic systems. To address this, the article investigates the repeatability and reliability of these testing procedures through an interlaboratory comparison approach.

In the field of collaborative robotics safety, several studies have focused on aspects such as human comfort prediction. A recent work has proposed in [15] a multi-linear regression-based model to predict human comfort levels in human–robot collaboration (HRC) scenarios, accounting for multiple comfort factors simultaneously. While this model demonstrates high accuracy in predicting comfort levels, it does not address the crucial issue of the forces that need to be applied by the robot. To ensure human safety, it is essential to implement systems that respect safety standards, such as ISO/TS 15066, to regulate force application during real-time interactions with robots.

Another study, in article [16], presents a methodology for the asymmetric measurement of collision forces and pressures in collaborative robots, with a focus on their time-dependent behavior. The methodology was validated through experimental measurements aimed at determining the robot’s collaboration level based on force and pressure at various speeds and distances from the base. The results indicated that collision forces increase as the robot approaches its base, with speed being the key factor influencing collision force values. The study highlights the importance of designing robotic movements to minimize human contact and suggests that the robot’s speed could be a limiting factor in industrial environments with strict cycle time requirements. However, the limitations of the study include the use of relatively low impact speeds (up to 400 mm/s), which may not fully reflect the conditions of fast-paced industrial applications. Furthermore, the study does not specifically address the need to comply with safety standards such as those set by ISO/TS 15066, which regulate the forces and pressures a robot can safely exert on humans. It is crucial to also consider which part of the human body is impacted to ensure that the robot’s movements are compliant with these safety regulations.

Safety is a crucial topic in many industrial and scientific fields. The study of forces and the use of specific sensors, such as Tek Scan (T-Scan) and FPF, are widely applied in various applications. These technologies enable the monitoring and analysis of pressure and force distribution through post hoc analysis, providing reliable solutions to ensure durability and prevent structural damage or wear. In the medical field, for instance, a thin-film pressure transducer system was compared with the FPF technique to measure tibio-femoral contact areas in total knee arthroplasty (TKA) devices. Results showed that T-Scan provided 11–36% higher values than FPF, highlighting its superior reliability, as developed in [17].

Similarly, the work in [18] present Flexiforce sensors that have been employed to study impact force distribution in helmet testing, offering precise mapping of forces under different thermal conditions.

In dentistry, T-Scan and FPF were used to analyze occlusal forces on teeth affected by non-carious cervical lesions (NCCL), demonstrating a strong correlation between heavy occlusal forces and lesion progression, as presented in [19].

The study [20] compares the performance of two pressure measurement systems, FPF and T-Scan, in estimating area, force, and pressure. Both systems were tested by applying known forces to sensors placed between a cylindrical peg and a steel base plate. FPF data were analyzed using two methods: the erase method, which manually removes areas outside the stain, and the threshold method, which analyzes only the stained pixels. The T-Scan system, which uses matrix-based sensors data acquisition, proved to be more accurate than FPF, especially for estimating area and pressure. The study highlights the importance of evaluating sensor technology for precise measurements.

Additionally, recent studies in material science have emphasized the importance of capturing spatially distributed stress responses under dynamic loading conditions. For instance, in this study [21] the authors investigated mechanochemical activation in triblock copolymers, showing how chain topology and spatial stress distribution critically affect material behavior. Such insights further underscore the need for high-resolution, real-time force sensing technologies in robotic applications, where understanding local stress responses is equally vital.

Furthermore, advanced material-based sensor systems have been explored in the literature to enhance flexibility and multifunctionality. For instance, a recent review [22] discusses tactile and thermal sensors built from carbon nanomaterial-filled polymer composites (CNPCs), emphasizing their potential in soft and curved surfaces, such as robotic skins. These materials offer intrinsic adaptability, although challenges remain regarding integration and standardization in robotic safety contexts.

Building upon these materials, a recent dissertation [23] developed and validated a systematic design methodology for CNPC-based tactile sensors specifically tailored to human-collaborative robots (HuBots). The work compared coating and dispersion methods for embedding multi-wall carbon nanotubes (MWCNTs) and demonstrated the advantages of the coating technique for constructing adaptable soft sensor networks. A prototype tactile sensing system was successfully integrated and tested on a HuBot platform, further confirming the feasibility of CNPC-based solutions in real-world collaborative robotics. Among biodegradable and biocompatible materials, silk fibroin (SF) has gained attention as a promising candidate for flexible pressure sensor applications due to its natural piezoelectric properties, compatibility with human tissue, and environmental sustainability. Recent research has explored the structure, piezoelectric characteristics, and fabrication methods of SF-based sensors, including capacitive, resistive, triboelectric, and piezoelectric types, with promising applications in personalized medicine and human–robot interaction [24]. Another recent advancement in the field of flexible pressure sensing involves the development of a highly sensitive capacitive sensor based on a porous ionic membrane [25]. The device consists of a polyvinyl alcohol/potassium hydroxide (PVA/KOH) dielectric layer sandwiched between two indium tin oxide-polyethylene terephthalate (ITO-PET) electrodes. Its sensing mechanism relies on the formation of electric double layer (EDL) capacitance at the electrode-dielectric interfaces. By engineering a porous microstructure in the dielectric layer, the sensor achieves both a wide sensing range and a remarkably high sensitivity (up to 20.83/kPa), while maintaining fast response times (50 ms) and bending insensitivity. A sensor array prototype was also fabricated, enabling the spatial mapping of pressure distributions, which holds significant promise for applications in wearable electronics and robotic tactile systems. A complementary approach involves the integration of piezoelectric materials with micro-structured elastic substrates to enhance sensitivity to gentle touches. A recent study proposed a thin-film piezoelectric pressure sensor that incorporates a polydimethylsiloxane (μ-PDMS) layer to mimic the sensitivity of human skin [26]. The sensor does not require an external voltage source, unlike traditional FET-based solutions, and is capable of detecting low-pressure stimuli with high fidelity. The microstructure enhances deformation under light contact, resulting in a threefold increase in sensitivity compared to flat-layer configurations. The device exhibits a wide dynamic range (0.23–10 kPa) and fast signal relaxation, making it particularly suited for biomedical palpation and real-time force monitoring in collaborative robotics.

These studies reveal the widespread use of such sensors but also highlight their limitations, particularly in applications requiring real-time monitoring and repeatability. Our proposed solution addresses these limitations exposed above using an embedded system that simulate human body interaction with collaborative robots that integrates FSR, load cells, and a real-time data acquisition system based on an FPGA. Furthermore, the entire embedded system is integrated with VSM with adjustable stiffness, creating the VSITD. Unlike sensors like FPF, this system offers real-time monitoring of forces and pressures, ensuring higher precision and repeatability in dynamic environments. This combination allows the system to adapt and test various parts of the human body, fully complying with the ISO/TS 15066 standard for collaborative robotics safety. The VSITD, along with the embedded system, ensures long-term pressure measurements representing the data with high precision thanks to the FSR sensor and shown in 2D map and instantaneous force measurements with high accuracy via load cells. The integration of these components not only guarantees precise force and pressure monitoring but also facilitates dynamic, real-time adjustments, making it particularly suited for applications in collaborative robotics and beyond.

## 3. Mechanical Structure

This section presents the concept and design of the VSM, which adjusts stiffness through the rotation of symmetrically connected springs. By modifying the rotation angle, the system achieves a wide stiffness range, ensuring adaptability and precise control. The design focuses on material selection, key component integration, and dynamic adjustment capabilities.

The force-displacement relationship and the corresponding stiffness variations are analyzed based on the initial rotation angle. The system exhibits distinct stiffness behaviors, ranging from near-zero stiffness to a maximum when the springs are fully horizontal.

A synchronized rotation mechanism ensures the desired stiffness curve, utilizing a vertical slider guided by a leadscrew and inclined slots. The relationship between slider displacement and spring rotation angle is examined to provide insights into the stiffness adjustment process.

### 3.1. Springs Design

**Figure 1:**
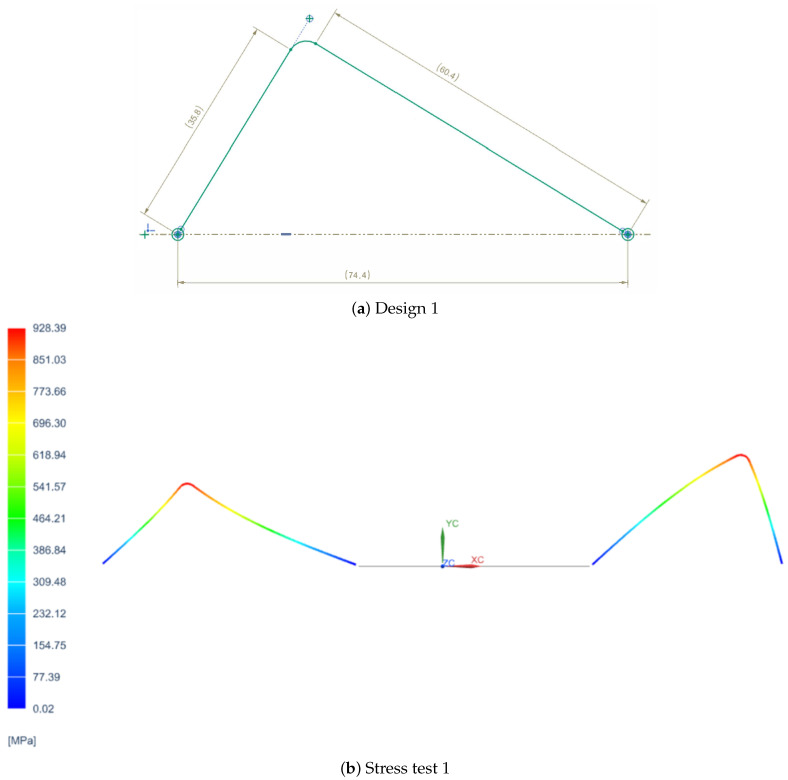
First spring configuration and its stress analysis.

**Figure 2:**
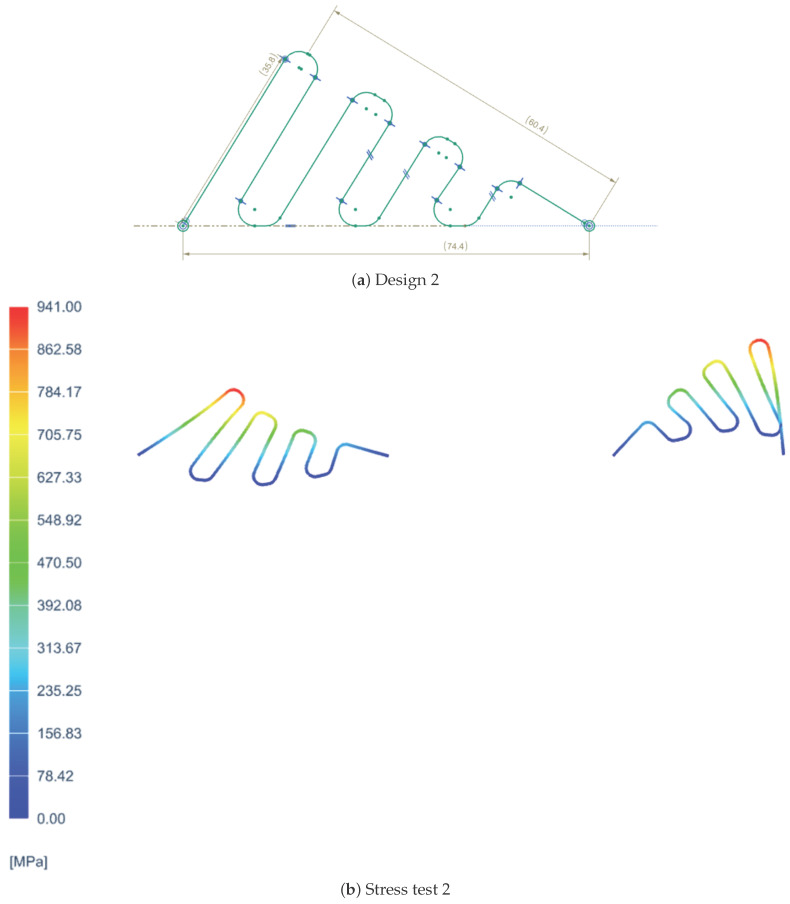
Second spring configuration and its stress analysis.

**Figure 3:**
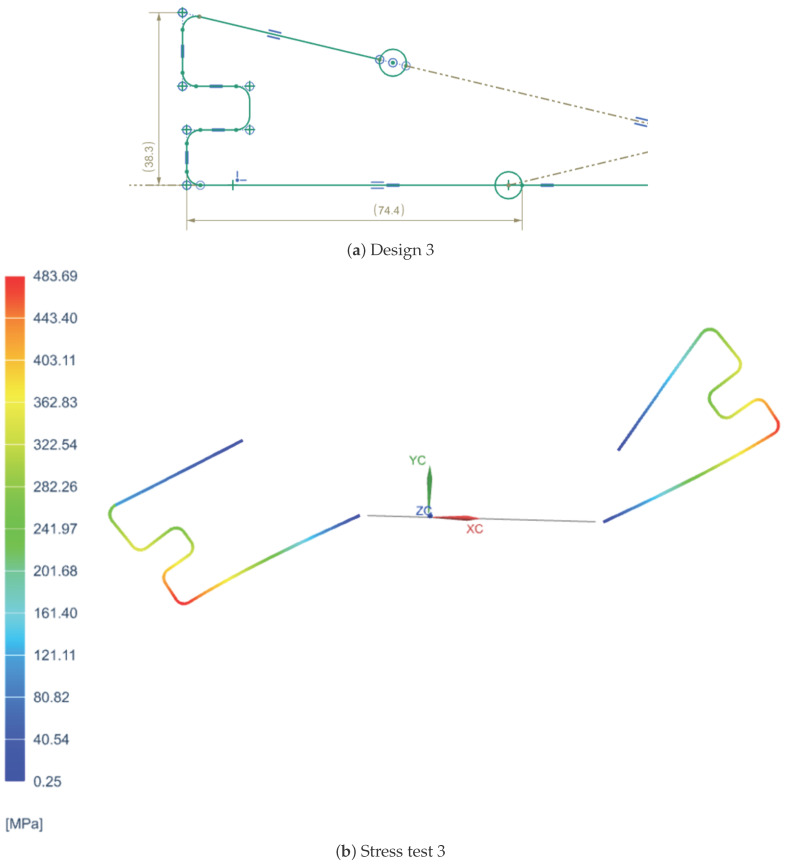
Third spring configuration and its stress analysis.

In order to determine the most suitable spring design for the VSM, three different configurations were tested, each with distinct geometric characteristics. The first design featured a simpler shape with fewer curvatures, as shown in Figure 1a, prioritizing ease of fabrication. The second design introduced a greater number of curves to enhance flexibility and extend the stiffness variation range, as shown in Figure 2a. However, the second design featured curves that were too tight to be physically realizable due to manufacturing limitations. The third design, on the other hand, did not include many curves but proposed a different type of curvature, as depicted in Figure 3a.

Each spring was subjected to a series of tests to evaluate its mechanical behavior, focusing on stiffness performance, stress distribution, and deformation under load. Additionally, manufacturability constraints were considered, as overly tight curvatures, as seen in the second design, would pose significant challenges in production. Feedback was collected from various companies specializing in precision spring manufacturing to assess the feasibility of each design.

**Figure 4:**
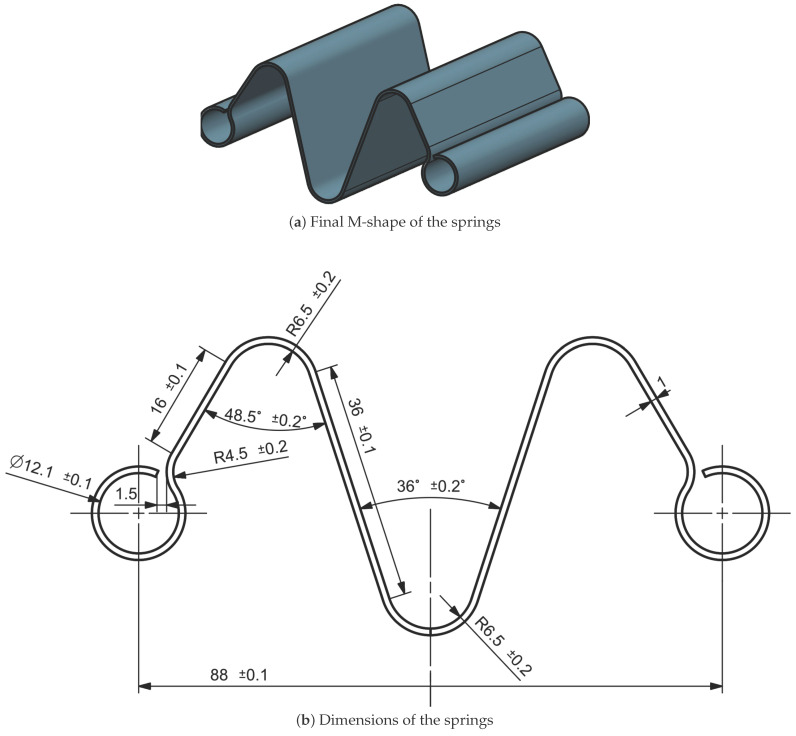
Configuration chosen for the springs utilized in the VSM. In (**a**) is shown the 3D design of the spring and in (**b**) are shown the dimensions of the spring.

Based on the test results and industry feedback, the final spring design was chosen as a compromise between the first and second configuration. The second design, with its tight curvatures, was discarded due to the production difficulties. Therefore, the curves from the second design were reduced to two, forming an “M-shaped” spring as shown in Figure 4, which provided an optimal balance between performance, manufacturability, and cost-effectiveness.

Additionally, Figure 1b, Figure 2b, and Figure 3b representing the stress distribution in MPa at various points of the spring for each of the three configurations were included for a more detailed analysis of their behavior under load. These are constructed using specific materials chosen for their mechanical properties: carbon steel C67s/75s tempered and temperable, as well as stainless steel AISI 301 and AISI 316L.

Among the candidate materials analyzed—tempered carbon steel C67S, temperable carbon steel C75S, and stainless steels AISI 301 and AISI 316L—the final prototype was manufactured using C67S. This material provided an optimal balance between elastic stiffness, fatigue resistance, and industrial feasibility. Is also important to specify that during the design of the spring, the width and the length of the springs have been evaluated.

As a representative case, the finite element method (FEM) is employed to analyze the deformation of a spring aligned at 0° with respect to the horizontal axis under applied loading. This configuration serves as a benchmark scenario commonly adopted for the validation of elastic deformation models. The simulation was conducted using a nonlinear approach, considering progressive time increments up to 10 s. The results show an increase in deformation over time, with the maximum displacement growing from 1.153 mm in the first frame to 6.607 mm in the last analyzed frame. Table 1 reports the minimum and maximum displacement values at key frames of the simulation. The reported frames correspond to only 4 out of the 21 frames available in the full FEM analysis.

**Figure 5:**
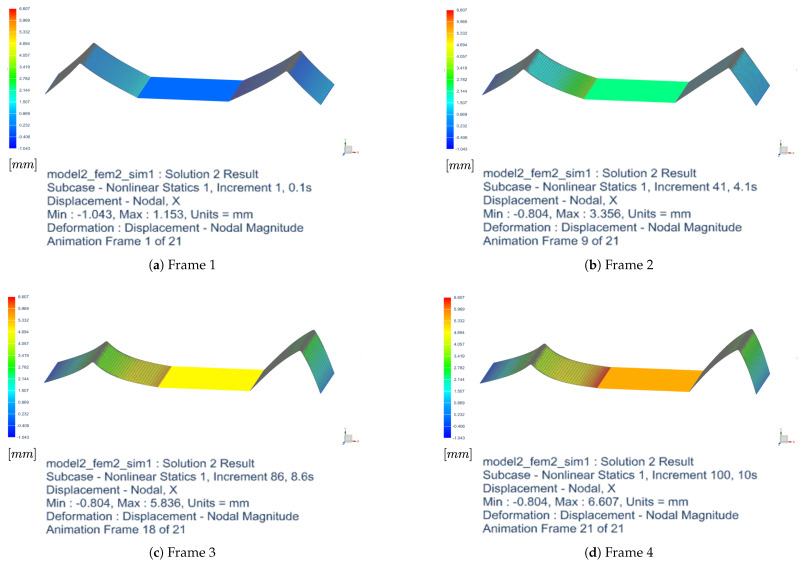
Deformation of the spring at different frames. The first row shows Frame 1 (**left**) and Frame 2 (**right**), while the second row shows Frame 3 (**left**) and Frame 4 (**right**).

Figure 5 illustrates the deformation of the spring at different time increments. The analysis highlights a nearly linear initial deformation trend, followed by a subsequent slowdown toward an asymptotic value. The minimum displacement remains unchanged from Frame 9 onward, suggesting the presence of a constraint or a less stressed region of the spring.

### 3.2. VSM Design

The VSM is based on the concept of stiffness variation through mechanical adjustment. Typically, it involves two springs that are symmetrically connected and rotated to vary the stiffness of the system. This is crucial in applications like human–robot collaboration, where the interaction force needs to be adaptable depending on the context of the task or the sensitivity of the human–robot interaction.

**Figure 6:**
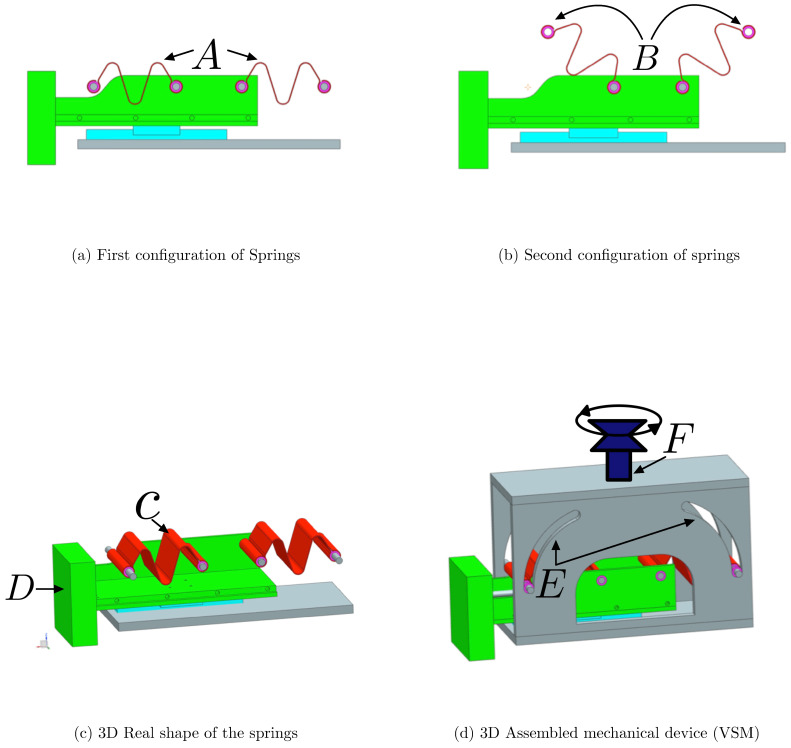
Mechanical structure of the Variable Stiffness Mechanism (VSM). In Figure (**a**,**b**) the letters represent the following: (**A**,**B**): the end positions of the springs, indicating their rotation range; in figure (**c**) are shown with letter (**C**), the 3D model of the curved springs used in the system and with (**D**) the surface where FSR matrix and load cells are installed; in figure (**d**) is shown with letter (**E**) the slots through which the bearings slide to vary spring angle and stiffness and with letter (**F**) the vertical slider actuated via a leadscrew to control the symmetric rotation of the springs.

**Figure 7:**
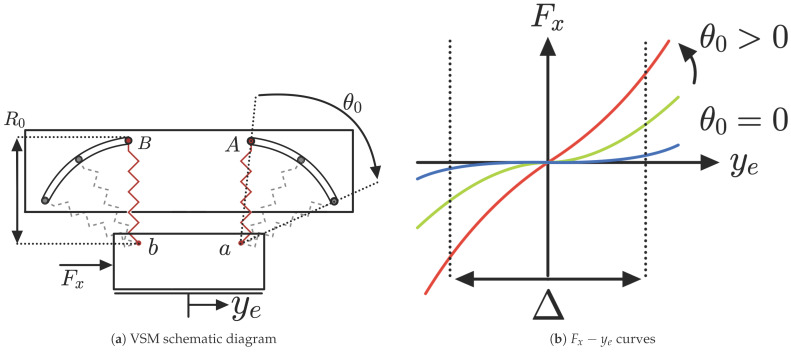
(**a**) VSM schematic diagram. (**b**) $F_{x}-y_{e}$ curves considering both springs contribute.

In particular, the mechanical composition of the VSM is illustrated in Figure 6, which provides a schematic overview of the entire system. The components are labeled (A–F) to clearly identify the spring terminals, the slider mechanism, the surface for sensor placement, and the actuation method. The regions marked as *A* and *B* indicate two distinct configurations of the springs, allowing variation in the angle from $0^{\circ }$ to its maximum. The 3D model of the springs is indicated with *C*, where the springs are shown with their specific shape. These are constructed using specific materials: carbon steel C67s/75s tempered and temperable, as well as stainless steel AISI 301 and AISI 316L. The surface denoted by *D* is designed to host the FSR sensors and the load cells, which are interposed between two metallic plates to measure forces accurately. The slot labeled E shows the track where the bearings are connected to the springs slide, thereby varying the stiffness of the mechanism. Finally, F indicates the slider that enables this movement, allowing for precise control over the stiffness adjustment. The implementation of a VSM requires the use of two springs: specifically, two identical springs, with stiffness $K_{s}$ each one, connected to the output slider at points *a* and *b*, as shown in Figure 7. The other ends (points *A* and *B*) of these springs are linked to two rollers sliding within respective circular slots. The radius of the circular slots is $R_{0}$ and their centers of curvature are located at points *a* and *b*. These circular slots permit the springs to freely rotate without inducing deformation when at their natural length $R_{0}$.

### 3.3. Stiffness Analysis

Initially, both springs are set at the same rotation angle $\theta_{0}$ from the vertical position. An external force $F_{x}$ applied to the output slider produces a displacement $y_{e}$, while the positions of points *A* and *B* remain unchanged. The stiffness behavior, represented by the $F_{x}-y_{e}$ curves, depends on $\theta_{0}$. When $\theta_{0}=0^{\circ }$, the springs are vertically aligned, resulting in nearly zero stiffness. As $\theta_{0}$ increases, the system exhibits positive stiffness, reaching a maximum of $2K_{s}$ when $\theta_{0}=90^{\circ }$ with horizontal springs.

In the actual mechanical implementation, $\theta_{0}$ is adjusted via a vertical slider mechanism that symmetrically displaces the spring supports, producing a controlled variation in the initial angle. The system is designed to operate within a practical angular range of $\theta_{0}\in \left[0^{\circ },80^{\circ }\right]$, which ensures safe modulation of stiffness without compromising the structural integrity of the springs.

The $F_{x}-y_{e}$ curves remain approximately symmetric around the origin, with variations depending on the displacement magnitude. The combined effect of both springs determines the overall stiffness response, where one spring compresses and the other extends, influencing the stiffness variation. An operational region Δ is defined, within which the variation in the zero-stiffness curve remains negligible.

**Figure 8:**
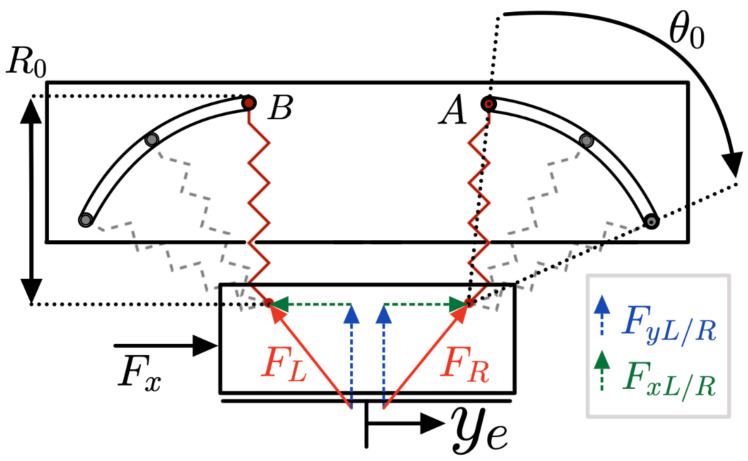
Schematic of the VSM considering both springs.

Figure 8 shows the force diagram of the VSM. The forces are depicted with components along the Cartesian x and y planes for both the left and right springs. The displacement has been denoted as $y_{e}$ and the rotation of the springs as $\theta_{0}$.

The force and stiffness characteristics of the VSM are derived based on the displacement-force relationship and the rotation angle of the springs. The force expressions are illustrated in Equations (1) and (2):

(1) $$F_{R}=K_{s}{\left[{\left(R_{0}\;sin\;\theta_{0}-y_{e}\right)}^{2}+{\left(R_{0}\;cos\;\theta_{0}\right)}^{2}\right]}^{1/2}-R_{0}\;\left[N\right]$$

(2) $$F_{L}=K_{s}{\left[{\left(R_{0}\;sin\;\theta_{0}+y_{e}\right)}^{2}+{\left(R_{0}\;cos\;\theta_{0}\right)}^{2}\right]}^{1/2}-R_{0}\;\left[N\right]$$

Through these, it is possible to analyze the respective horizontal components, expressed in Equations (3) and (4).

(3) $$F_{xR}=\frac{\left(R_{0}\;sin\;\theta_{0}-y_{e}\right)}{{\left[{\left(R_{0}\;sin\;\theta_{0}-y_{e}\right)}^{2}+{\left(R_{0}\;cos\;\theta_{0}\right)}^{2}\right]}^{1/2}}*F_{R}\;\left[N\right]$$

(4) $$F_{xL}=\frac{\left(R_{0}\;sin\;\theta_{0}+y_{e}\right)}{{\left[{\left(R_{0}\;sin\;\theta_{0}+y_{e}\right)}^{2}+{\left(R_{0}\;cos\;\theta_{0}\right)}^{2}\right]}^{1/2}}*F_{L}\;\left[N\right]$$

The external force $F_{x}$ needs to balance $F_{x}R$ and $F_{x}L$ such as shown in Equation (5):

(5) $$F_{x}=F_{xL}-F_{xR}\;\left[N\right]$$

By differentiating this equation, the stiffness *K* is obtained, showing its dependence on the initial rotation angle $\theta_{0}$ and the spring stiffness $K_{s}$. The initial stiffness $K_{0}=2K_{s}\sin\left(2\theta_{0}\right)$ is theoretically maximized when $\theta_{0}=90^{\circ }$, but this value exceeds the mechanical design limits of the current prototype.

In practice, the system reaches its maximum effective stiffness at around $\theta_{0}=80^{\circ }$, which is the upper bound allowed by the current VSM geometry.

### 3.4. Configuration of the Rotation Angle Parameter

**Figure 9:**
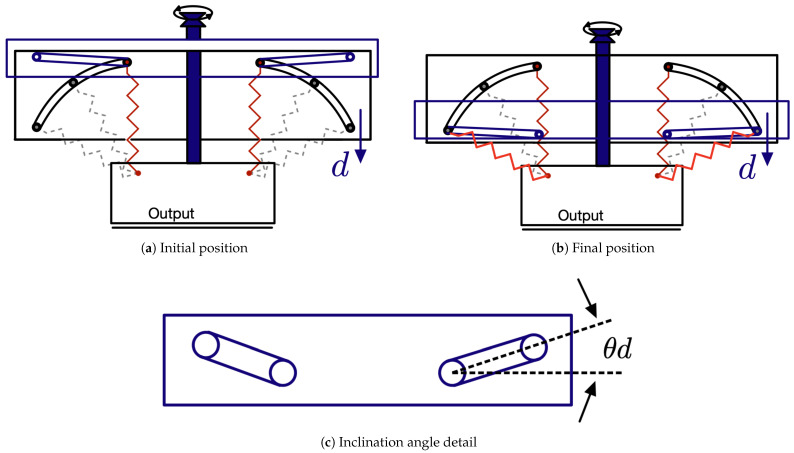
Using a vertical slider makes it possible to change the rotation $\theta_{0}$. Figure (**a**) shows the initial position, Figure (**b**) the final position and Figure (**c**) the angle of incline.

To achieve the desired stiffness curve, it is essential for both springs to undergo simultaneous rotation by the same angle $\theta_{0}$. This synchronized rotation is facilitated by the introduction of a vertical slider, as depicted in Figure 9. The vertical slider is equipped with two identical inclined straight slots, guiding the rollers attached to the ends of the springs. The initial position is illustrated in Figure 9a. Through the rotation of a leadscrew drive, the vertical slider can be vertically displaced by an amount *d*. In the Formula (6) is shown the relationship between *d* and $\theta_{0}$:

(6) $$d=R_{0}\left(1-cos\theta_{0}\right)+\phi \;tan\theta_{d}\;\left[m\right]$$

Consequently, the rollers traverse the slots, resulting in the concurrent rotation of both springs.

The complete expressions representing the VSM and the relationship between $F_{x}$ and $y_{e}$ and its rotation angle parameter $\theta_{d}$ can be found in [1].

## 4. Embedded System Design

This chapter explores the integration of the VSM with advanced force measurement systems, combining a Force-Sensitive Resistor (FSR) matrix and load cells to achieve detailed pressure mapping and precise force measurements. It delves into the design and implementation of a dedicated Printed Circuit Board (PCB) for interfacing with the FPGA, ensuring reliable data acquisition through signal amplification, digitization, and stable transmission. Additionally, the role of operational amplifiers in conditioning sensor signals is analyzed, optimizing them for ADC conversion. The circuit simulation in LTSpice-17.2.4validated the system’s performance, ensuring accurate measurements for advanced applications such as human–robot interaction and tactile simulation.

### 4.1. VSITD Concept

VSITD integrates a VSM with a FSR matrix, load cells, and the FPGA. Such a system provides valuable data on the distribution of the applied pressure on the sensor surface, enabling a more localized and detailed understanding of interaction forces. This is essential for applications where precise feedback is required, such as simulating the interaction between a robot and human body. The force measurement is based on the change in resistance, and the information derived can be processed to create a dynamic map of pressure, providing insight of the regions where the strongest contact has occurred.

On the other hand, load cells are designed to measure the magnitude of the force exerted on a given object. Unlike FSRs, load cells are more suited for capturing the total force at the point of application, offering high precision in force measurement at any given moment. This makes load cells particularly effective in scenarios where instantaneous force measurements are needed, such as in robotics for detecting the strength of a robot’s grip or determining the force of impact during a collision. The load cells convert the mechanical force into an electrical signal, which is proportional to the force applied. The key advantage of using load cells in a VSM lies in their ability to provide direct and instantaneous readings of force, critical for systems that need to react immediately to changes in the interaction force. The load cells, in particular, are piezoelectric sensors from PCB Piezotronics and their signals were acquired using a dedicated analog signal chain. Specifically, the outputs from the load cells were first amplified by the multi-purpose signal conditioner 482C from PCB Piezotronics and then acquired by the analog to digital converter DC2395A by Linear Technology, coupled to the data acquisition module DC890B from Analog Devices. This process ensured proper signal amplification and filtering. This setup allowed for accurate and dynamic measurement of force values applied to the system.

The FSR configuration used is current-to-voltage converter, commonly referred as transimpedance amplifier (TIA). This offers a more uniform and ideal transfer function compared to traditional voltage dividers. Unlike voltage dividers, a TIA allows a fixed voltage to be applied across a single FSR element, regardless of the presence of other parallel FSRs or resistances in the circuit.

By applying the ideal operational amplifier assumptions to the circuit in question, the voltage across the input terminals is actually zero, i.e., $V_{IN-}=0$ V (a virtual ground). Consequently, the input terminals of the operational amplifier do not source or sink any current, meaning that the current through the feedback resistor $\left(I_{RF}\right)$ is equal to the current through the FSR $\left(I_{FSR}\right)$.

From this point, the calculations become straightforward, and the output voltage $\left(V_{\mathrm{OUT}}\right)$ is given by the following equation:

(7) $$V_{\mathrm{OUT}}=-\frac{V_{\mathrm{DRIVE}}}{R_{\mathrm{FSR}}}\times R_{F}\;\left[V\right]$$

In the equation, $V_{\mathrm{OUT}}$ denotes the output voltage of the resistive divider, $V_{\mathrm{DRIVE}}$ is the applied supply voltage, $R_{\mathrm{FSR}}$ represents the variable resistance of the FSR that changes with applied force, and $R_{F}$ is the fixed resistor used in the circuit.

FSR sensors, comprising two layers of conductive material separated by a non-conductive layer, detect variations in force by changing their electrical resistance when pressure is applied. Their primary strength lies in their sensitive and precise detection of pressure variations. However, their responsiveness may be influenced by factors such as temperature, humidity, and environmental conditions, potentially requiring proper calibration for optimal functionality.

**Figure 10:**
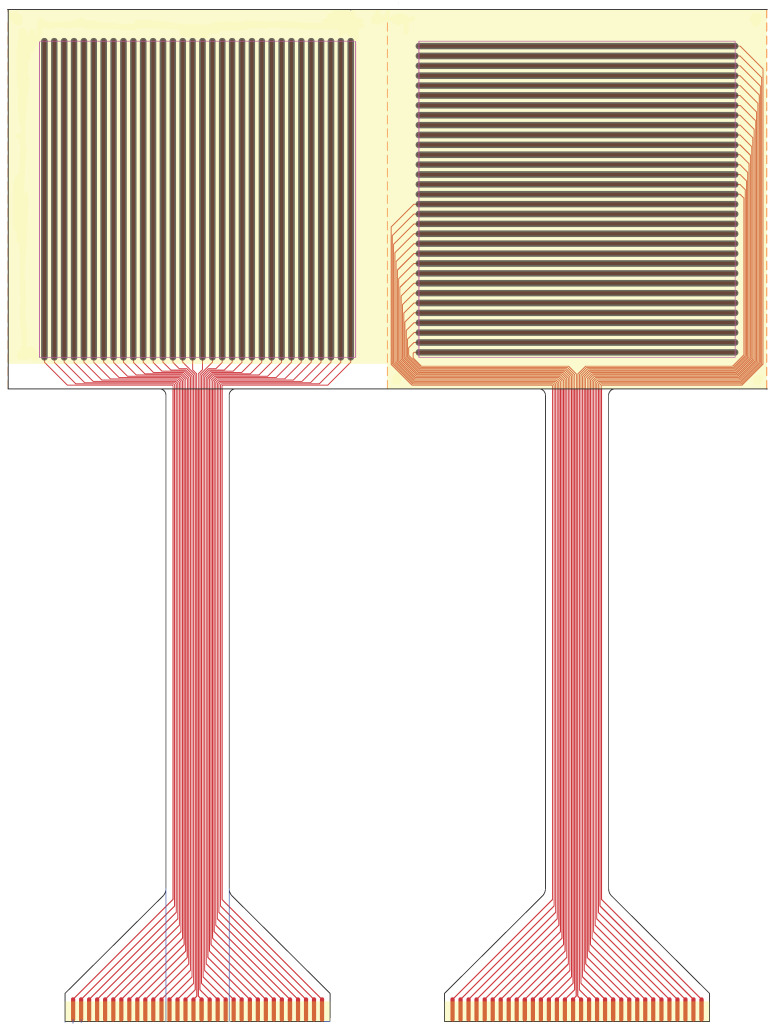
FSR matrix layers.

The specific sensor employed is a 32 × 32 grid array composed of FSRs spanning a surface area of 10 × 10 cm. This custom configuration, as shown in Figure 10, involves leveraging the properties of these materials to detect pressure along two distinct axes. One layer is structured to form a grid of columns sensitive to force along the x-axis. Simultaneously, the other layer, placed orthogonally, forms rows sensitive to force along the y-axis. Their intersection points form individual force sensors capable of detecting pressure applied at specific coordinates in the matrix.

The material used in the FSR matrix is XactFSR, a proprietary force-sensitive polymer developed by Sensitronics. It is based on PET (Polyethylene Terephthalate), which serves as the substrate for the conductive and pressure-sensitive layers. This material offers improved linearity, stability, and repeatability under repeated loading, making it well-suited for high-resolution pressure sensing applications in dynamic environments.

Pressure at a specific point is measured by detecting changes in voltage or current across the sensor located at the intersection of a particular row and column. These variations indicate the applied pressure at that position. The calibration procedure described in Section 5 ensures accuracy, and data processing converts sensor readings into meaningful pressure information at specific points within the matrix.

This matrix configuration enables the creation of a 2D map of pressures applied to a given surface.

This process typically includes applying known pressures to specific points within the matrix and adjusting sensor outputs to match these expected values. Calibration compensates for variations in sensor sensitivity, environmental factors, or manufacturing tolerances that might affect accuracy.

Calibration links the actual pressures applied to the sensor readings. This process creates a calibration curve, which is then used to convert sensor readings into accurate pressure values.

Furthermore, ongoing recalibration might be necessary to account for changes in environmental conditions, sensor degradation over time, or alterations in the sensor matrix setup. Regular recalibration ensures the continued accuracy and reliability of the pressure measurements within the matrix.

### 4.2. PCB Design for FSR Integration via FPGA

To enable the accurate reading and processing of data from the custom FSR matrix sensor, a dedicated PCB was designed. This PCB plays a crucial role in interfacing FPGA with the force sensor and managing the acquisition of sensor data.

This ensures reliable signal transmission, amplification, and digitization of the sensor outputs. By using the FPGA, the system can quickly process the FSR data and perform tasks such as force measurement, pressure mapping, and dynamic adjustment in real-time. This configuration provides the required precision and speed for simulating human–robot interactions, where timely feedback and high accuracy are critical.

**Figure 11:**
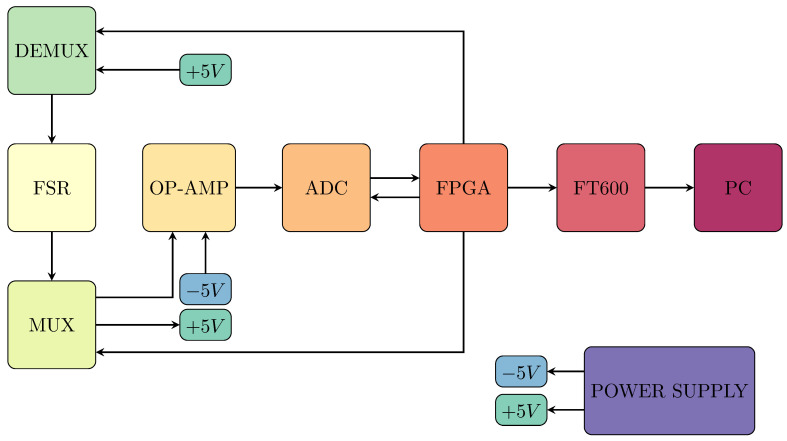
Block diagram of the system to read data from the FSR sensor.

Figure 11 shows the block diagram of the system components to read and to analyze data from the FSR sensor.

In more detail, the Analog-to-Digital Converter (ADC) used is the LTC2311-14, a high-performance 15-bit Successive Approximation Register (SAR) ADC manufactured by Linear Technology (now part of Analog Devices). This ADC is designed for high-resolution applications and supports differential input voltages up to ±4 V. The LTC2311-14 converts the analog signal into a 15-bit digital value, which includes a sign bit for precise bipolar signal conversion.

The ADC operates by capturing the analog signal through a Sample-and-Hold (S/H) circuit, which ensures a steady voltage level during conversion. The SAR architecture is used to iteratively approximate the analog input value, providing accurate digital outputs. The SAR ADC’s high resolution makes it suitable for applications requiring precise analog-to-digital conversion.

The multiplexers used, ADG708BRUZ are 8-channel analog CMOS multiplexers by Analog Devices, with 4 units operating in MUX mode and 4 in DEMUX mode. Key features of the *ADG708BRUZ* include its eight-input configuration, which enables the selection of one channel out of eight, the use of three digital address lines ($A_{0}$, $A_{1}$, $A_{2}$) for input selection, and a fast switching time of 14ns, allowing for rapid signal transitions.

Linear voltage regulators are employed to ensure stable output voltages for sensitive electronic components. Specifically, the project uses the following Low Dropout Regulators (LDOs):

- The ADP7142AUJZ-5.0-R7 from Analog Devices is used to provide a stable +5 V output with low noise. Its input voltage range is from +6 V to +40 V, making it versatile for various power supply scenarios. The device offers very low noise, with $V_{rms}$ noise of 10μV and a peak-to-peak noise of 10μV, ensuring clean power for sensitive electronics.
- The ADP7182AUJZ-5.0-R7, also from Analog Devices, is used to provide a stable −5 V output with low noise. The input voltage range spans from +5.5 V to −28 V. Its $V_{rms}$ noise is 18μV, and the peak-to-peak noise is 18μV, ensuring reliable negative voltage regulation for precision applications.

These LDOs are chosen for their ability to deliver precise voltage regulation with minimal noise, making them ideal for powering components that require stable and clean power in both positive and negative voltage scenarios.

The LT1819CMS8-PBF operational amplifier from Linear Technology (now part of Analog Devices) is used. This high-speed op-amp is selected for its ability to operate with both single and dual power supplies, making it flexible for different circuit designs.

The LT1819CMS8-PBF’s ability to handle a dual power supply configuration ensures that there are no saturation issues at the ADC input, making it the ideal choice for maintaining signal integrity in this project.

The integration of operational amplifiers with the ADC (LTC2311-14) was evaluated through a simulation conducted in LTSpice-17.2.4. The LTC2311-14 operates within a bipolar input range of ±4.0965 V, and the op-amps were configured to appropriately condition the signals before they are fed to the ADC.

The data acquisition system for the FSR matrix is designed to support high-throughput sampling using the FTDI FT600 USB 3.0 FIFO interface, which allows data transfers up to 200 MB/s. The analog signals from the FSR matrix are digitized using the LTC2311-14 15-bit SAR ADC, operating at a nominal sampling rate of up to 5 Msps. Due to the multiplexed scanning configuration required for the 32×32 matrix, the effective sampling rate per active sensing element is approximately 10–20 kHz, depending on the number of rows and columns enabled during acquisition. The switching logic for the multiplexers is controlled by a Xilinx Artix-7 FPGA, which ensures precise channel selection and synchronization. The typical latency introduced by the acquisition pipeline—from analog input to digital data availability on the host PC is below 1 ms.

### 4.3. Operational Amplifier Configuration

**Figure 12:**
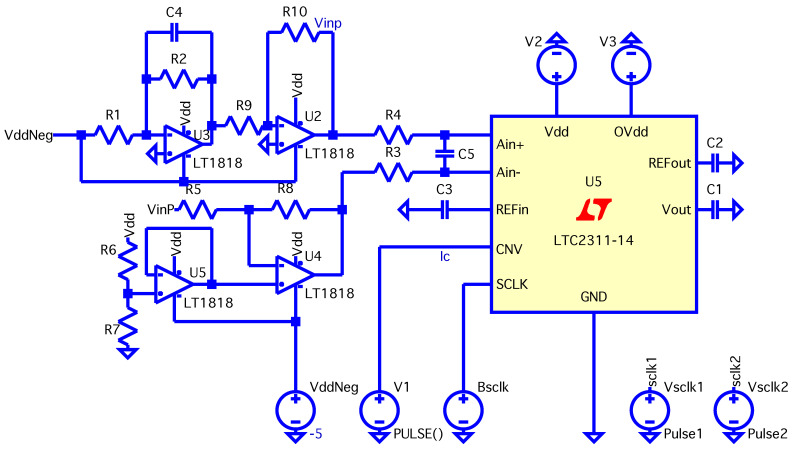
Simulated circuit in LTSpice-17.2.4.

Four operational amplifiers were used ($U_{2},U_{3},U_{4},U_{5}$), consolidated into two integrated components using the LT1819CMS8-PBF model, as depicted in Figure 12. The configuration of the amplifiers is summarized as follows.

- $U_{2}$: Inverting op-amp with negative unity gain. This configuration inverts and amplifies the signal as per the equation:
  $$V_{out}=-V_{in}\;\cdot \;\frac{R_{10}}{R_{9}}\;\left[V\right]$$
  with $R_{9}=R_{10}=200\;\Omega$.
- $U_{3}$: Inverting transimpedance Op-Amp. This stage converts the current from the FSR matrix into a voltage. The feedback resistor $R_{2}$ is chosen to prevent saturation, based on the minimum resistive value of a FSR sensor. The output voltage is given by
  $$V_{out}=-V_{dd}\;\cdot \;\frac{R_{2}}{R_{1}}\;\left[V\right]$$
  where $R_{1}$ is the inherent resistance of the FSR, and $V_{dd}$ is the supply voltage.
- $U_{4}$: Differential op-amp configuration amplifies the difference between two input signals, ensuring proper conditioning for the differential inputs of the ADC. The output voltage is calculated as follows:
  $$V_{out}=V_{in}^{+}\;\cdot \;\left(1+\frac{R_{8}}{R_{5}}\right)-V_{in}^{-}\;\cdot \;\frac{R_{8}}{R_{5}}\;\left[V\right]$$
  Simplified, this equation yields:
  $$V_{out}=2V_{in}^{+}-V_{in}^{-}\;\left[V\right]$$
  with $R_{8}=R_{5}=200\;\Omega$.
- $U_{5}$: Non-inverting buffer provides a reference voltage for the subsequent stages. The input is derived from a voltage divider composed of resistors $R_{6}$ and $R_{7}$, and the output voltage is given by
  $$V_{out}=V_{dd}\;\cdot \;\frac{R_{7}}{R_{6}}\;\left[V\right]$$
  where
  $$\begin{array}{cc} \; & R_{6}=200\;k\Omega \\ \; & R_{7}=120\;k\Omega \\ \; & V_{dd}=5\;V \end{array}$$

The operational amplifier stages are designed to fully exploit the voltage range allowed by the differential inputs of the LTC2311-14 ADC, which can handle inputs ranging from ±4.1V. The output from $U_{2}$ and $U_{3}$, when reaching the ADC, is conditioned by the lower stages (U5 and U4) to guarantee the voltage stays within the proper range for ADC input. The correlation between the outputs of the op-amps and the differential inputs of the ADC is given by the following:

$$A_{in}=A_{in}^{+}-A_{in}^{-}$$

The ADC’s differential input can accept a maximum voltage difference of ±4.1V, with a saturation margin of approximately ±0.1V.

The entire circuit was simulated using LTSpice-17.2.4 to verify the performance of the operational amplifier configurations and obtain proper signal conditioning before ADC input.

## 5. Experiments and Results

**Figure 13:**
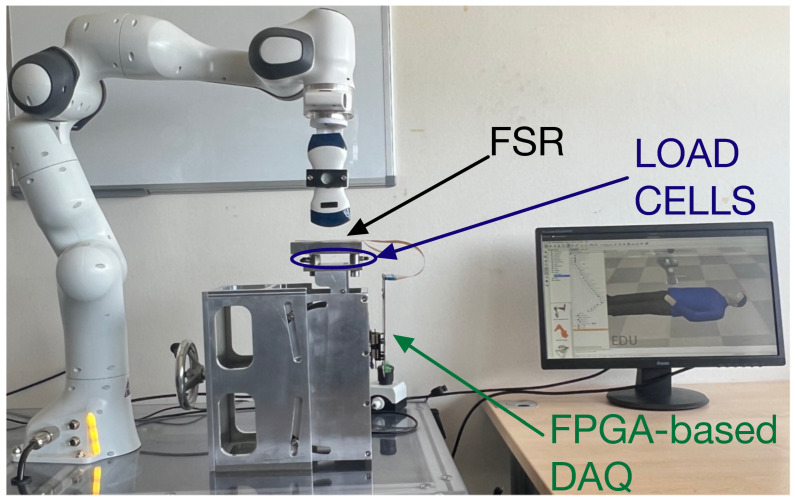
Experimental setup that can be used to calibrate the robotic arm for controlled force and pressure application in compliance with ISO/TS 15066.

An illustrative application, for example, in collaborative robotics consists of calibrating a robotic arm to ensure that it maintains a consistent contact force and pressure on a specific region of the human body. This calibration step is essential to prepare the robot for tasks requiring controlled interaction, such as performing an ultrasound examination. By verifying that the applied forces comply with the ISO/TS 15066 safety standard, this process enables safe and repeatable physical contact between the robot and the human operator. The experimental setup designed for this purpose is shown in Figure 13.

This section presents the experimental setup and the results obtained in the project. Section 5.1 details the experiments carried out to calibrate the VSM device using a mechanical press.

It is important to highlight that the FSR sensor was specifically designed for this project, with the goal of covering a surface area of the VSM measuring 10×10 cm^2^. For the following experiments, the sensor was initially produced without any pre-calibration. This initial calibration is a crucial step to ensure optimal sensor performance. Additionally, for the static tests, the sensor was assumed to be uniform and perfectly flat, allowing for a consistent pressure distribution over its entire surface. Static tests refer to experiments in which constant time-invariant forces are applied to the sensor to evaluate its steady-state response under controlled pressure conditions. This assumption enabled a more controlled analysis of the force applied across the entire sensing area.

Several approaches were considered for implementing the static tests. The first idea involved using a “chamber at controlled pressure” to achieve a uniform gas pressure distribution over the entire sensor area. However, this approach was discarded due to the high cost of manufacturing such a device and the technical challenges associated with applying the required pressure levels.

The second approach considered was the use of an automated system, such as a CNC machine. In this scenario, the CNC would have been programmed to move precisely across each point of the 32×32 matrix and apply different pressure levels at each pixel, enabling a point-by-point calibration of the FSR. While this method would have been suitable in terms of achieving the required millimeter-level positioning accuracy, it was ultimately discarded due to the system’s inability to exert the necessary forces without risking mechanical damage. This limitation is primarily due to the extremely small sensing area of a single pixel in the FSR matrix, which is approximately $10^{-5}$ mm^2^.

Section 5.2 presents the chosen approach, which consists of calibrating a selection of pixels from different regions of the matrix to analyze their characteristics. The final outcome of this process is a fitting model that characterizes the sensor’s response.

Finally, in Section 5.3, once the sensor’s response had been determined, dynamic impact tests were conducted using soft human body parts, such as human fingers, to observe pressure variations over time. The recorded values were then compared with a high-precision force sensor, the F/T Nano 17, to assess the accuracy and reliability of FSR measurements.

### 5.1. VSM Prototype and Stiffness Experiment

To validate the design, prototypes of the VSM were constructed and tested under real-world conditions. This experiment simulates the dynamic forces that a robot might encounter during its interaction with a human, such as contact forces caused by intentional touches or undesired collisions.

A mechanical press was used to apply varying amounts of force onto the VSM, testing its response and ability to absorb or transmit these forces across different frequencies. This experiment helps in determining how effectively the VSM can handle real-world interaction dynamics.

Using the mechanical press, it was possible to perform tests on the VSM by applying different force levels per millimeter with the press moving downward at a velocity of 25mm/min. Data acquisition and subsequent graphical analysis enabled the collection of a sufficient number of samples to accurately reconstruct the system’s force–displacement response.

The data acquisition was performed using a custom system based on LabVIEW 2021 which automatically generates a .csv file. This file was then imported into Matlab_R2023b, where a dedicated script generated graphical representations of the results.

The tests conducted with the mechanical press were performed at various stiffness levels, approximately corresponding to the values specified by ISO/TS 15066 for different human body parts: 10N/mm, 25N/mm, 30N/mm, 35N/mm, 40N/mm, 50N/mm, 55N/mm, 60N/mm, and 75N/mm. Subsequently, an additional test was conducted to evaluate the mechanism’s functionality using a single spring element.

**Figure 14:**
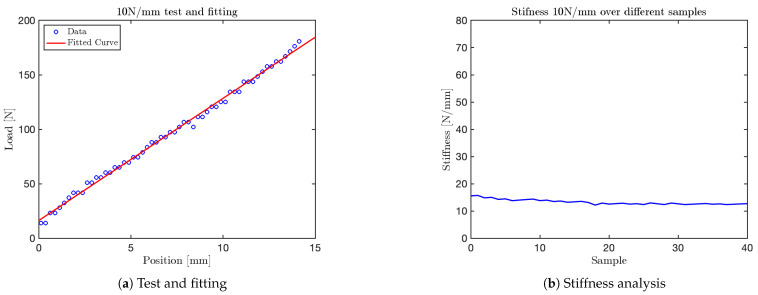
Results from the 10 N/mm test.

**Figure 15:**
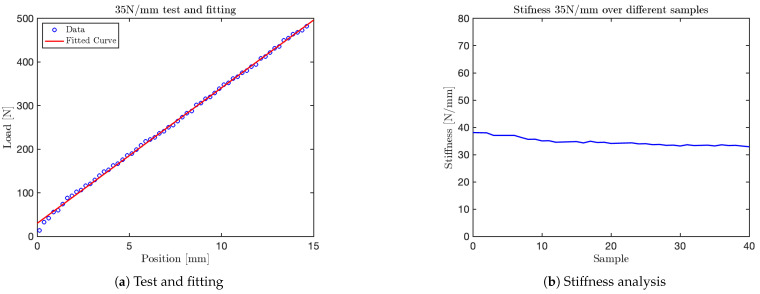
Results from the 35 N/mm test.

**Figure 16:**
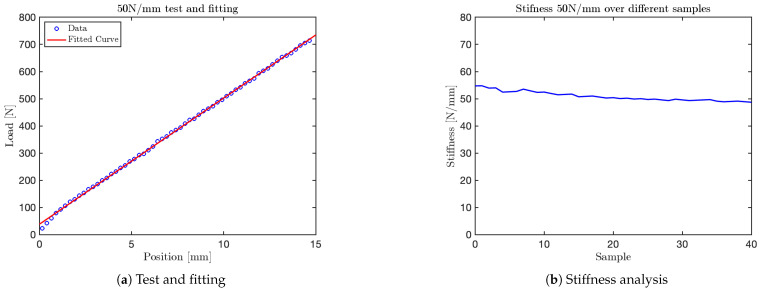
Test at 50 N/mm.

**Figure 17:**
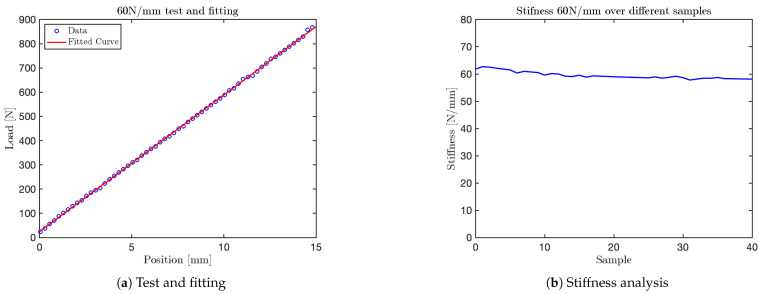
Test at 60 N/mm.

**Figure 18:**
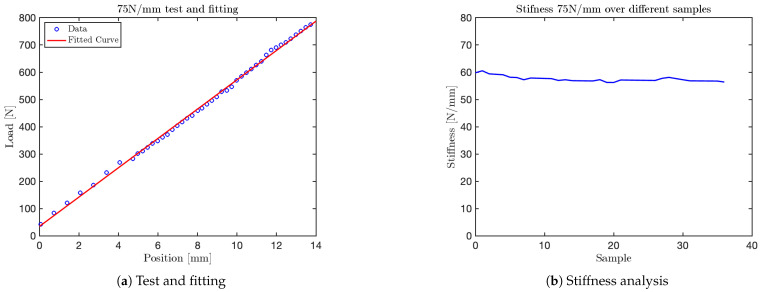
Test at 75 N/mm.

The graph obtained for the 10N/mm test is shown in Figure 14. Additional graphs for tests at 35N/mm, 50N/mm and 60N/mm are shown in Figure 15, Figure 16, and Figure 17, respectively. The final test, corresponding to the nominal stiffness of 75N/mm, showed saturation behavior and confirmed that the maximum effective stiffness of the VSM lies around 60N/mm, as illustrated in Figure 18.

It is important to note that the test conducted at a nominal stiffness of 75N/mm (see Figure 18) revealed a clear saturation in the system’s response. Specifically, while the initial portion of the force–displacement curve follows a linear trend, the stiffness begins to plateau beyond approximately 60N/mm. This indicates that the current VSM configuration is mechanically limited to an effective maximum stiffness slightly above this value. This range is sufficient to replicate the biomechanical characteristics of various human body regions—such as the head, arms, abdomen, and hands—whose equivalent stiffness falls within the 10–60 N/mm interval as specified by ISO/TS 15066. However, it does not fully encompass stiffer anatomical regions like the back, thigh, or shoulder, which may require values above 80 N/mm.

For each test, two types of plots are provided: subfigures labeled with (a) show the relationship between the applied load (y-axis) and the corresponding displacement (x-axis), as recorded by the force sensor integrated into the mechanical press. These plots provide a direct visualization of the VSM’s response to increasing displacement.

Subfigures labeled with (b) illustrate the evolution of stiffness—defined as the ratio between force and displacement—plotted against the sample index. In these plots, the y-axis represents estimated stiffness, and the x-axis represents the number of samples. The plots show that stiffness remains approximately constant throughout the application of the force, except for an initial transient caused by low initial contact force.

Importantly, each configuration of the VSM was tuned to match stiffness thresholds specified by ISO/TS 15066 for various body regions. By selecting an appropriate spring configuration that reproduces the desired stiffness (e.g., 10N/mm for the head, 35N/mm for the arm), the system guarantees that the forces generated during interaction do not exceed safe limits. This approach allows the VSITD to simulate compliant responses for specific human body parts, ensuring safety in accordance with the standard.

### 5.2. Static Tests

To ensure the correct functionality of the force sensor before performing the final impact tests between the human and the complete system (comprising the VSM, the FSR array with FPGA-based data acquisition, and the load cells), preliminary tests were conducted to calibrate the force sensor. These calibration tests were essential for verifying the sensor’s accuracy and reliability, ensuring that it provides precise measurements under operational conditions. A three-legged apparatus was designed and 3D printed to apply various weights and evaluate the response of the FSR sensors. The load distribution across the three legs was calibrated using load cells to ensure even pressure on the sensors. Three different leg supports were used:

- A support applying pressure to a single cell.
- A support applying pressure to four adjacent cells.
- A cylindrical support for covering multiple cells.

This setup was crucial for verifying the linearity of the sensors’ response and identifying any saturation points. Data were transmitted to Matlab_R2023b via the serial port, where they were processed and organized into 2 × 2 matrices, a single cell, and the entire 32 × 32 matrix representation.

#### Characterization Results

**Figure 19:**
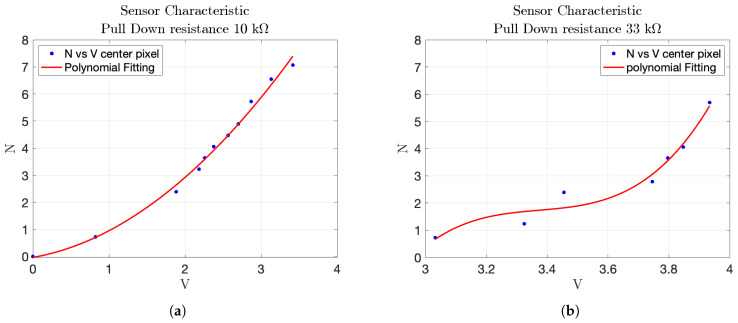
Comparison of polynomial response curves for different pull-down resistances. (**a**) Polynomial response with 10kΩ pull-down resistor. (**b**) Polynomial response with 33kΩ pull-down resistor.

**Figure 20:**
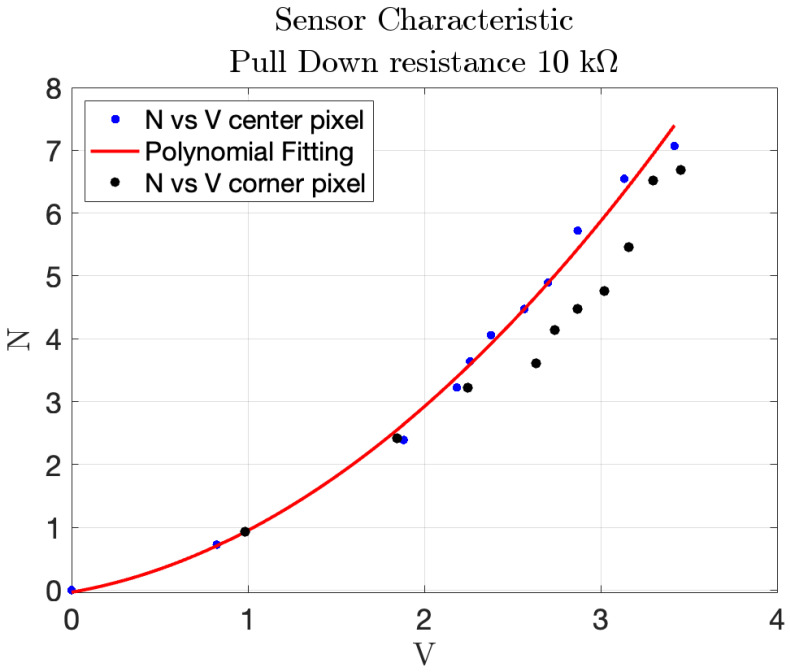
Polynomial response of the central sensor over different samples indicated by blue color, with scatter data from a corner cell with a black color.

**Figure 21:**
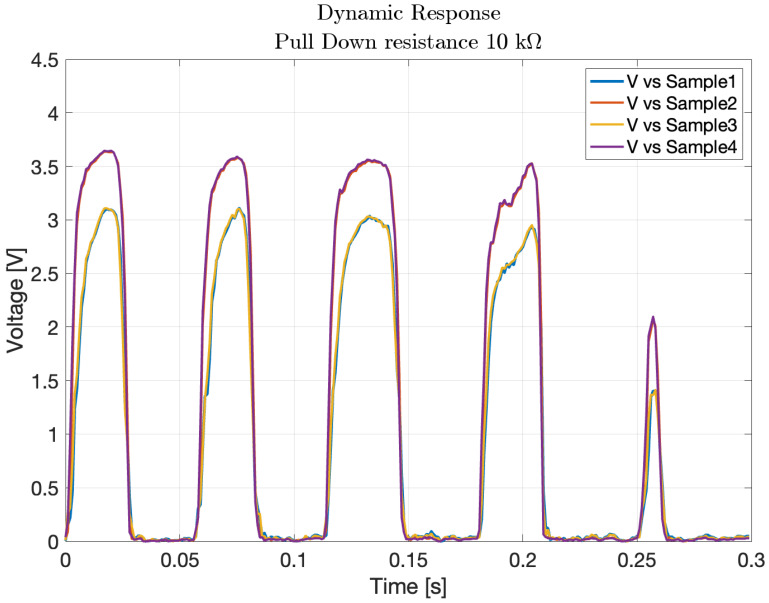
Dynamic response of four FSR cells during impact testing.

**Figure 22:**
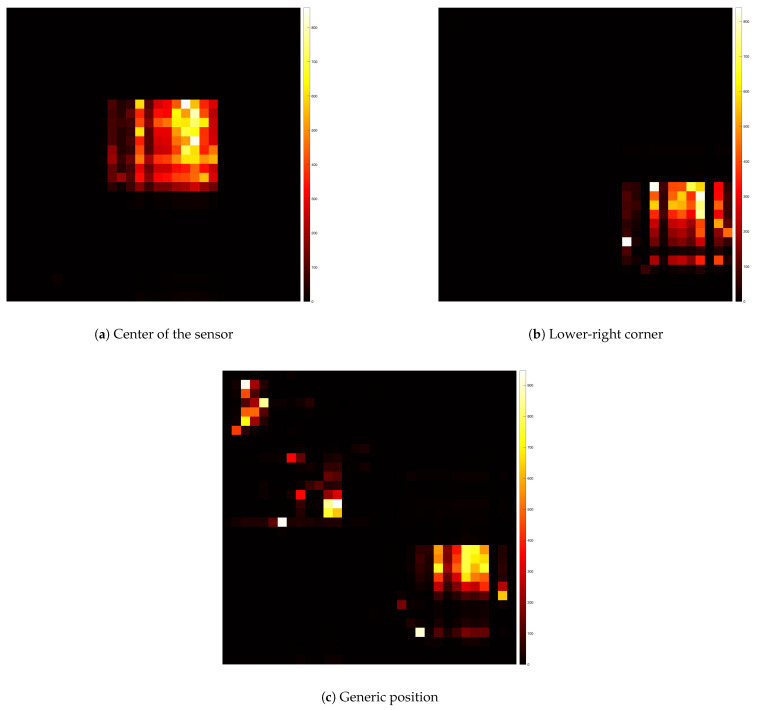
Pressure distribution measured by the matrix sensor at different positions: (**a**) center, (**b**) lower-right corner, and (**c**) generic location.

- Effect of pull-down resistor on sensor response: the first experiment investigated the influence of different pull-down resistors (10 kΩ, 22 kΩ, 33 kΩ, 2.7 kΩ) on the sensor’s response. The sensor readings, along with the corresponding polynomial fitting curves, were analyzed. A low pull-down resistor value resulted in minimal voltage variation with changes in weight, while very high values introduced significant non-linearity. The 10 kΩ pull-down resistor provided the most linear and appropriate response, tracking the increase in weight accurately. The equation of the polynomial fitting found with this test is
  (8) $$F=p_{1}\;\cdot \;v^{2}+p_{2}\;\cdot \;v$$
  where *F* is the measured force and *v* is the corresponding voltage. The associated root mean square error of the polynomial fitting is rms=0.1549 N. The obtained fitting coefficients were $p_{1}=0.4197$, $p_{2}=0.4928$, and they were subsequently used to convert the sensor’s output into pressure values during the impact tests.
  The results are shown in Figure 19a,b.
  Testing different pull-down resistor values is a fundamental step during the initial calibration phase of the sensor, as it allows identifying the optimal electrical configuration that maximizes the sensitivity and linearity of the output signal. Although the test with the 2.7 kΩ resistor was conducted, its results are not reported in the plots due to the rapid saturation of the output voltage. This configuration caused the sensor to reach its maximum output value with minimal applied force, making it unsuitable for capturing a wide range of pressures. Similarly, the 22 kΩ resistor was omitted from the final plots because its response curve was nearly indistinguishable from that of the 33 kΩ resistor, providing redundant information.
- Comparison of different points in the matrix: the second experiment compared the response of sensors located at the center and corner of the matrix. The characteristic function for the central sensor, derived using the 10 kΩ pull-down resistor, was plotted and compared with the data from the corner sensor. The comparison, shown in Figure 20, helped assess whether the sensor at the corner followed the same response pattern as the central sensor, providing insight into the uniformity of sensor performance across the matrix. In particular, the blue markers represent data from the central sensor used for curve fitting, while the black markers refer to the corner sensor. This comparison highlights the response uniformity of the matrix, confirming that even sensors located at the corners follow a similar trend to the central ones.
- Dynamic response to impact: the third experiment involved a 2 × 2 matrix of four adjacent force sensors subjected to five distinct impacts. The resulting data, shown in Figure 21, exhibited clear peaks corresponding to the force applications. This demonstrated the sensors’ ability to detect dynamic impacts effectively, capturing their response to applied forces.
- The objective of this test was not to quantify the applied impact force or energy, but to verify the uniformity and repeatability of the dynamic response across four adjacent sensing cells. A consistent, non-quantified force was delivered via a rigid three-legged tool, ensuring simultaneous and uniform mechanical stimulation of the selected sensors. This configuration enabled a reliable assessment of temporal response consistency, thereby validating the integrity of the matrix under repeatable dynamic loading conditions. This experiment demonstrate how two cells have the same response to the impact test instead other two have different peaks, in particular higher. This explains that the FSR matrix should be calibrated singularly to have every single characteristic of the single pixel.
- Pressure distribution and neighboring cell response: the fourth experiment involved a 2D reconstruction of the sensor array, using Matlab_R2023b to visualize pressure distribution. A cylindrical base applied pressure across multiple cells at both the corner and center of the sensor, as shown in Figure 22a,b. Additionally, pressure from various weights was applied, as depicted in Figure 22c, allowing for analysis of how localized pressure affects the sensor under pressure and its surrounding cells.

### 5.3. Dynamic Tests with Impact

To assess the behavior of the force FSR under dynamic conditions, a series of impact tests were conducted using a soft part of the body as the impactor.

In order to estimate the pressure values from the raw sensor output, a fitting procedure was employed based on static calibration tests. The relationship between the sensor’s electrical response and the applied pressure was modeled using the polynomial function, whose coefficients were determined experimentally in static tests

**Figure 23:**
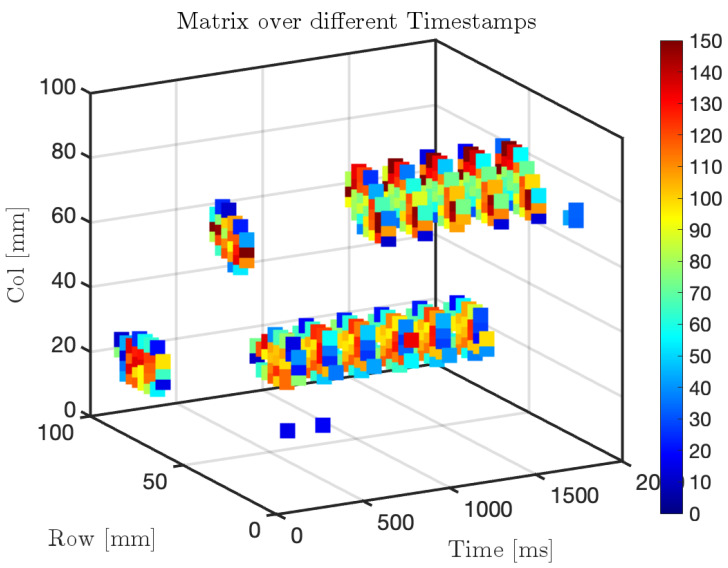
Three-dimensional visualization of pressure variation over time on the matrix sensor during repeated contacts.

Figure 23 presents the sampled pressure variations in a 3D representation across the entire matrix sensor, where only the points experiencing a significant pressure change are displayed using a scatter plot generated in Matlab_R2023b The results highlight how pressure values can reach relatively high levels (in bar), primarily due to the small contact surface of approximately 10 mm^2^ over which the force is applied. This behavior is expected, as pressure is inversely proportional to the contact area, meaning that forces applied on a reduced surface result in locally high-pressure values.

**Figure 24:**
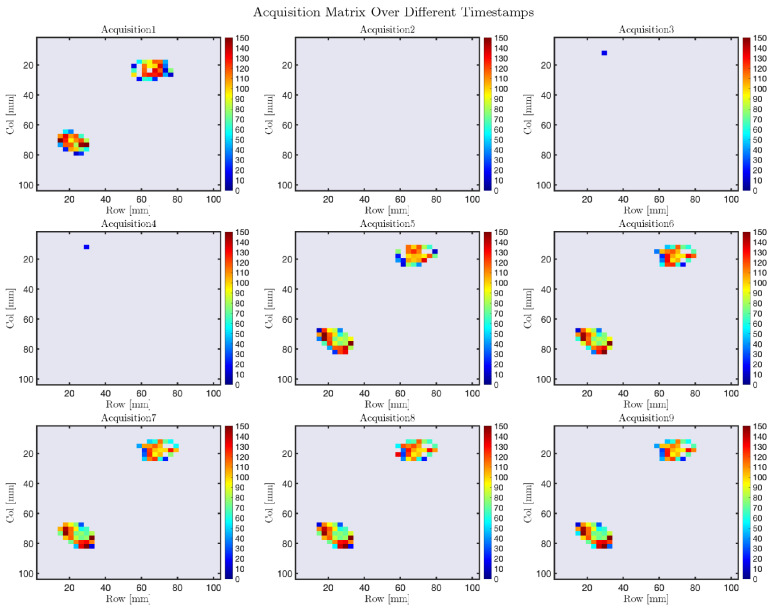
Two-dimensional representations of the pressure distribution acquired from the matrix sensor at different timestamps.

Figure 24 provides a 2D visualization of the individual matrix acquisitions, offering a clear representation of the pressure distribution over time. The graphical representation highlights spatial variations in pressure, where areas with values approaching zero indicate zones of no contact, while changes in color intensity reflect increasing pressure in specific regions. This visualization supports a detailed interpretation of how pressure evolves across the sensor matrix, emphasizing the dynamic behavior of the system during the acquisition phase.

To further validate the reliability and accuracy of the FSR sensor, a high-precision certified force sensor was employed as a reference. Specifically, we used the Nano 17 force/torque sensor from ATI Industrial Automation. This sensor, known for its precise and calibrated response, served as a benchmark for comparing the FSR readings.

The results confirmed that the FSR sensor consistently detected pressure variations, demonstrating good reliability. After conducting both static and dynamic tests on the FSR matrix, the data revealed that its response closely follows the behavior of the F/T sensor, further validating the accuracy of the measurements.

**Figure 25:**
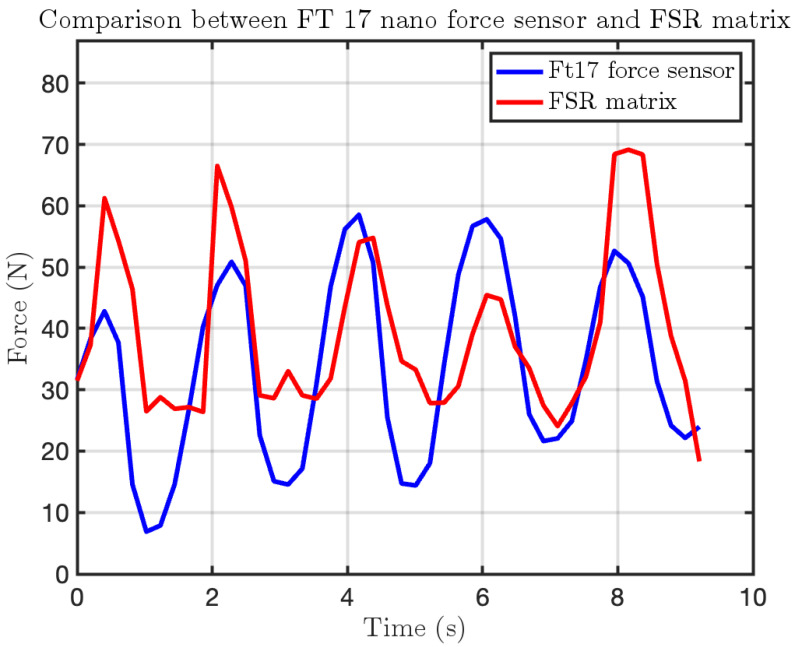
Impact test comparison using the FSR matrix and the F/T sensor Nano 17. The blue line represents the force measured by the Nano 17, while the red line corresponds to the force recorded by the active area of the FSR matrix.

Figure 25 illustrates this comparison, showing that the FSR sensor captures force variations in a manner similar to the F/T sensor. However, the values reported by the FSR sensor tend to be slightly higher on average, as it also accounts for the force applied by the F/T sensor placed above it. The plots further demonstrate that force impulses applied to the F/T sensor are detected by both measurement systems, reinforcing the consistency and reliability of the FSR matrix in tracking force variations.

## 6. Conclusions

In this work, we propose the development of the VSITD, a novel device aimed to validate human–robot interactive applications. This system, combining a 32 × 32 FSR matrix, FPGA-based data acquisition, and high-precision load cells, has demonstrated adaptability and safety, meeting the demanding requirements of collaborative robotics. Preliminary calibration tests confirmed the reliability and accuracy of the sensing elements. This calibration phase was crucial for verifying the consistency of measurements, enabling a reliable foundation for subsequent testing.

This rapid response allowed the system to simulate human-like biomechanical properties while the Robotic cell can test and ensure the safety and comfort of the interaction.

The integration of the FSR matrix and load cells proved to be a robust approach for understanding interaction dynamics. The FSR matrix provided high-resolution spatial data on pressure distribution, while the load cells delivered precise, point-specific force measurements. The hybrid sensing strategy enabled the VSM to capture a comprehensive dataset that informed real-time adjustments to its stiffness, enhancing both accuracy and functionality.

Looking ahead, the next steps for this project will involve the precise calibration of each cell in the FSR matrix to obtain an accurate sensor characteristic for the entire array. This will result in significantly more precise measurements. Moreover, a systematic study will be conducted to assess the impact response of the VSITD under varying velocities and angles of collision. This will enable a deeper understanding of the dynamic behavior under diverse operating conditions.

Additionally, the system will be tested in industrial environments, where the FPGA’s high-speed data acquisition capabilities, in conjunction with the PCB, will allow for live acquisition and real-time adjustments, further validating its potential for industrial applications. Such tests will focus on evaluating reliability under mechanical vibration, thermal changes, and electrical noise. Long-duration impact cycles and calibration drift will be analyzed to assess system robustness, with hardware adaptations implemented as needed.

To address the current stiffness limitation of approximately 60N/mm observed during experimental tests, future versions of the VSM will incorporate new mechanical solutions. These include the use of stiffer spring materials and revised geometries of the actuation mechanism. These improvements will extend the stiffness range to match even the most rigid human body parts. In conclusion, the VSM represents a significant innovation in collaborative robotics, combining advanced sensing technologies, real-time stiffness control, and strict compliance with international safety standards. It offers a fundamental testing tool aimed the calibration of robotic applications in healthcare, industrial automation, and beyond, where safety, adaptability, and natural interaction are critical. 

## Figures and Tables

**Table 1:** Evolution of the deformation of the spring at $0^{\circ }$ over time.

Frame	Time (s)	Min Disp. (mm)	Max Disp. (mm)
1	0.1	−1.04	1.15
9	4.1	−0.80	3.36
18	8.6	−0.80	5.84
21	10.0	−0.80	6.61

## Data Availability

Data is contained within the article.
